# Supplementary material for: Divergent mechanisms of steroid inhibition in the human ρ1 GABAA receptor
Source: Nat Commun. 2024 Sep 6;15:7795. doi: 10.1038/s41467-024-51904-7 (PMC11379708; doi:10.1038/s41467-024-51904-7)
Supplement: Supplementary file 1 — Supplementary Information [file 41467_2024_51904_MOESM1_ESM.pdf]

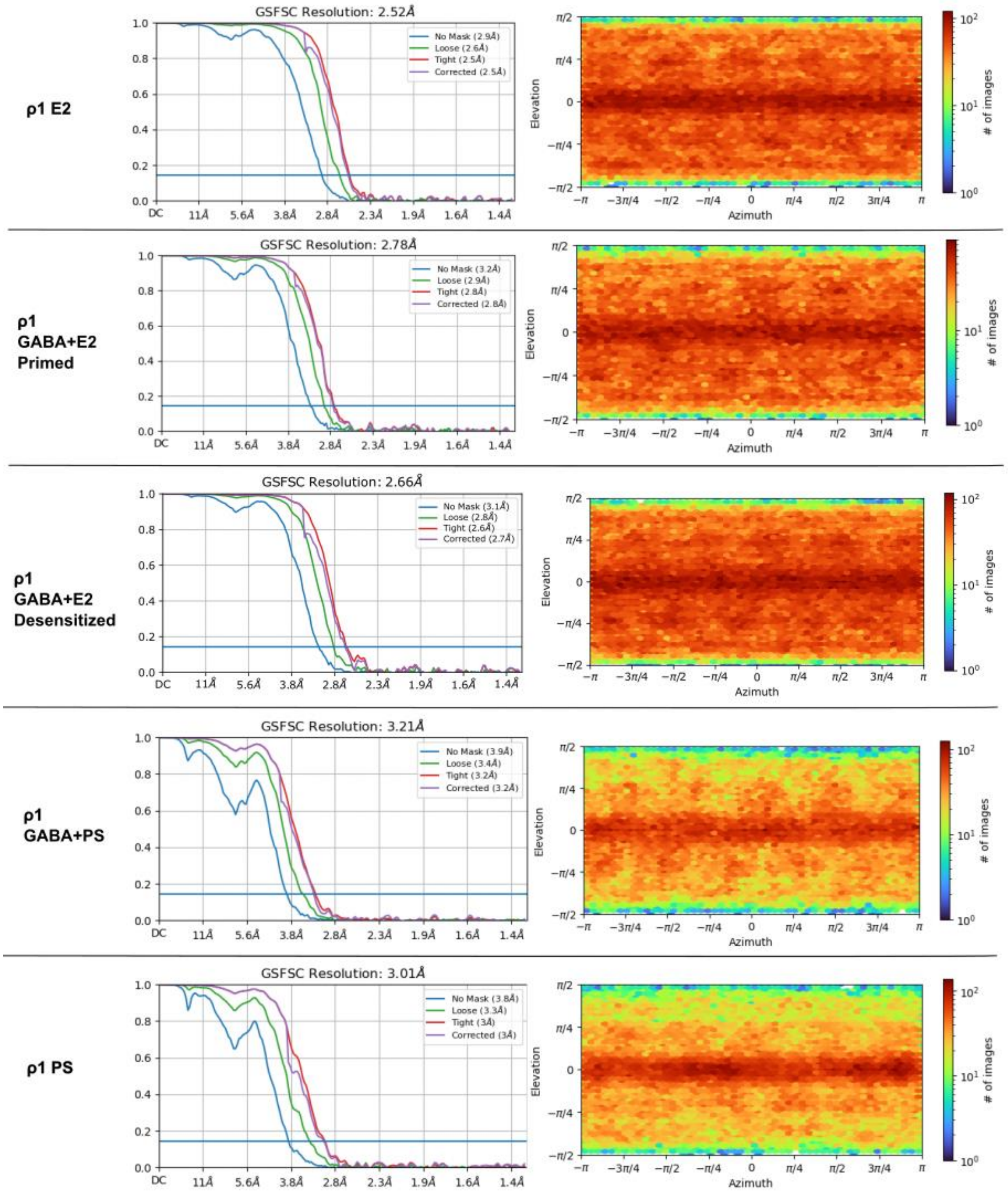

Supplementary Figure 1. Refinement plots for ρ1 structures with E2 and PS.

Left, Fourier shell correlations (FSC) versus overall resolution, including correlations for the unmasked map (blue) and with a relatively loose (green), tight (magenta), or corrected (purple) mask applied in CryoSPARC. Solid blue line represents gold-standard FSC (GSFSC, 0.143) indicator of true map resolution. *Right,* angular distribution of ρ1 particles in the presence of calcium, colored according to scale bar at right.

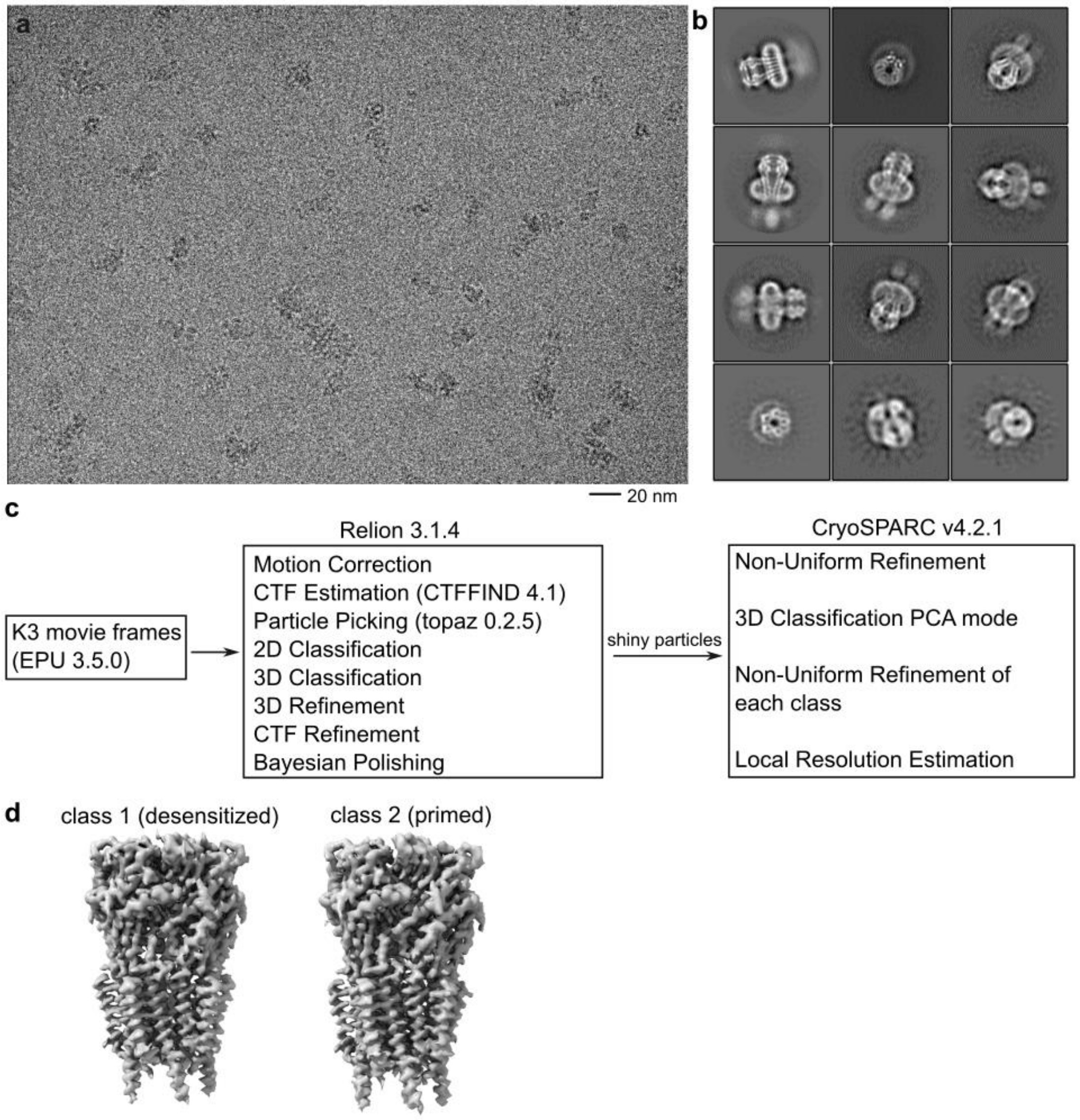

Supplementary Figure 2. Processing pipeline for ρ1-EM structures.

(a) Representative cryo-EM image from the dataset for ρ1-EM in the presence of E2.

(b) Representative 2D classification images from the dataset in A.

(c) Processing workflow for all datasets reported in this work.

(d) Representative 3D classification reconstructions from ρ1-EM dataset with GABA and E2.

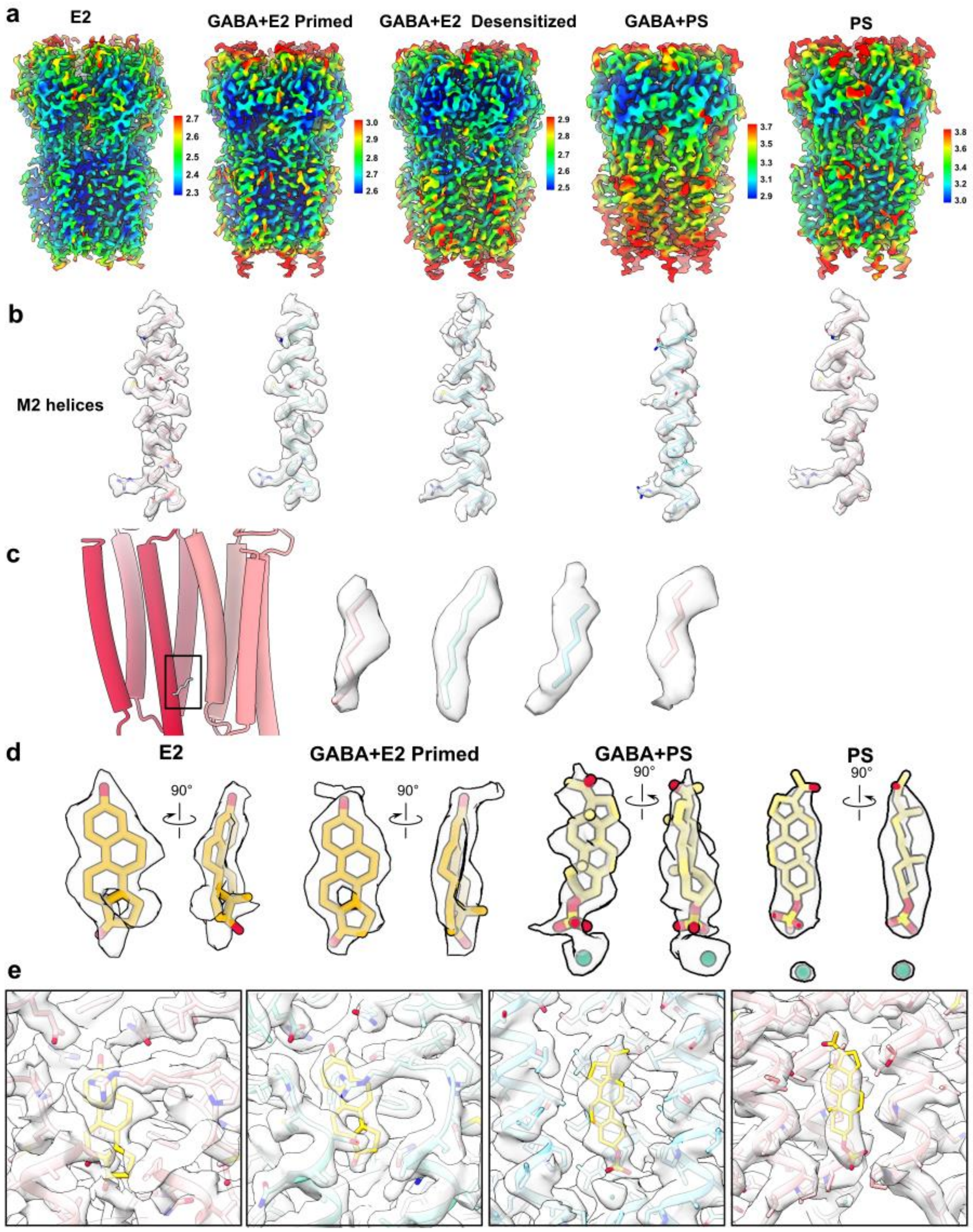

a
E2
GABA+E2 Primed
GABA+E2 Desensitized
GABA+PS
PS
b
M2 helices
c
d
E2
GABA+E2 Primed
GABA+PS
PS
90°
e

Supplementary Figure 3. Local resolution and representative densities in ρ1-EM.

(a) Cryo-EM maps reported in this study, determined in the presence of ligands indicated above each map, and colored by local resolution according to scale bars at right.

(b) Densities and models of M2 helices from all structures reported in this study, determined for ρ1-EM in the presence of (left to right) E2 alone, GABA+E2 in the primed state, GABA+E2 in the desensitized state, GABA+PS, and PS alone.

(c) Apparent lipid tail at the M3-M1(-) interface of several ρ1-EM structures, corresponding to the allopregnanolone binding site in the α1β2γ2 $GABA_A$ receptor. Leftmost cartoon shows a TMD interface between two subunits in the structure with E2, with the relevant lipid atoms as sticks. Maps and models at right show zoom views of lipid tails in structures determined with (left to right) E2 alone, GABA+E2 in the primed state, GABA+E2 in the desensitized state, and PS alone. No definitive lipid was observed in this region in the structure with GABA+PS.

(d) Densities and models associated with (left to right) E2 in the absence of GABA, E2 in the presence of GABA (primed state), PS in the presence of GABA, or PS in the absence of GABA. Each ligand is shown from two angles, with carbon in yellow, oxygen red, sulfur gold, and chloride ions green.

(e) Residues that surround each ligand shown in *d,* contoured at the same level as the corresponding ligand (yellow).

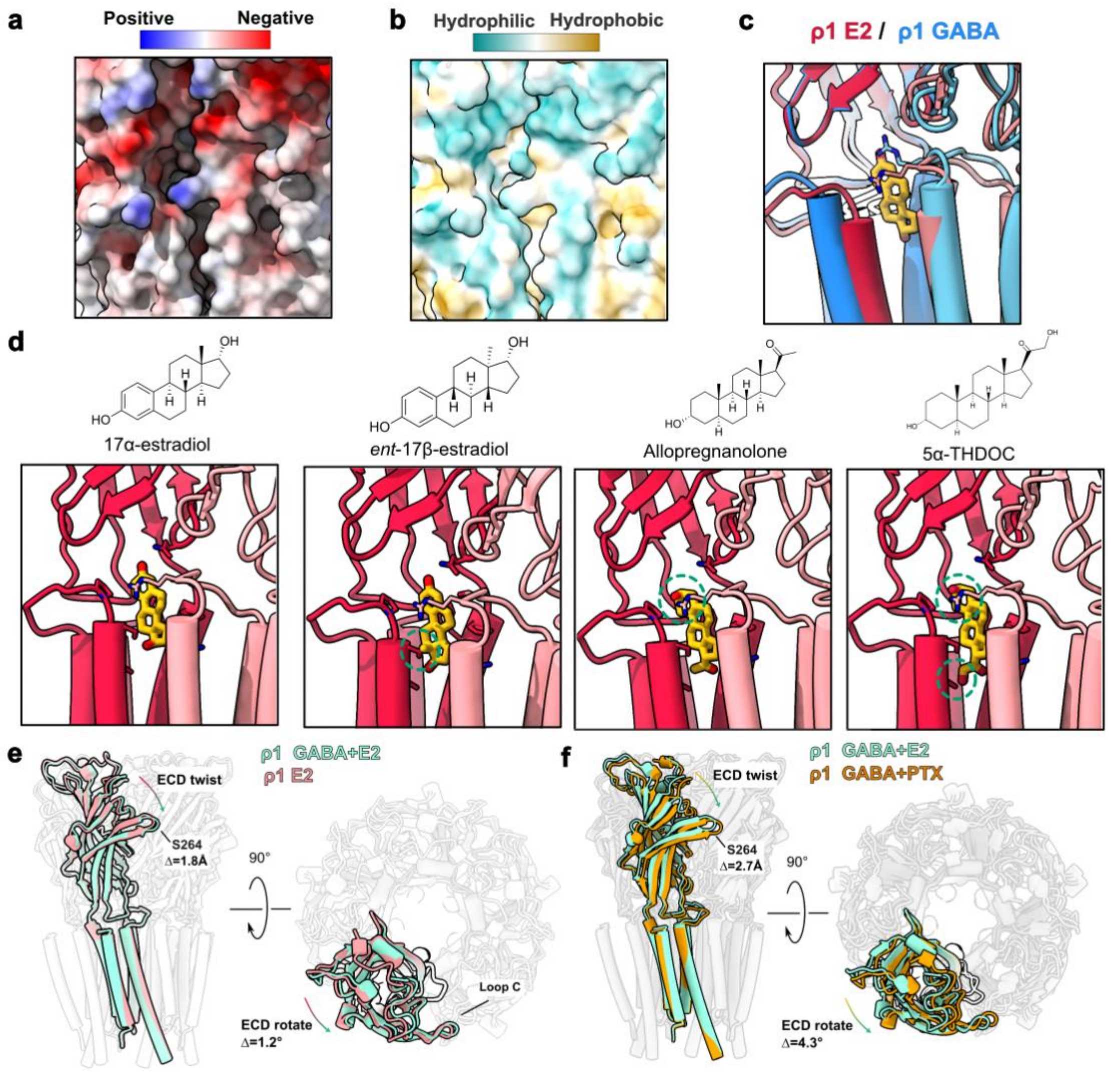

Supplementary Figure 4. Properties, selectivity and impact of E2 binding to ρ1-EM.

(a) Surface representation of a single E2 binding pocket, viewed as in *Figure 1f,* colored by electrostatic potential according to scalebar at top.

(b) Surface representation as in *a*, colored by hydrophobicity according to scalebar at top.

(c) Subunit interface as in *a*, showing a superimposition of ρ1-EM structures determined in the presence of E2 (protein in red, ligand in yellow) or GABA (blue). Conformational changes upon activation/desensitization alter the E2 site, likely precluding binding.

(d) Modeling of E2 homologs 17α-estradiol, ent-17β-estradiol, allopregnanolone and 5α-THDOC into a single interface of the structure with E2. Steroids are manually aligned on the E2 rings. Clashes between the protein and steroids are indicated by green circles.

(e) Superimposed structures with E2 alone (pink) and with GABA+E2 in the primed state (green), viewed from the membrane plane (left) or extracellular side (right). For clarity, all but one subunit is rendered semitransparent. Labels indicate relative translocation of the Cα atom of residue S264 (left), and rotation of a single ECD subunit (right), between the two structures.

(f) Superimposed structures of GABA-bound ρ1-EM with E2 in the primed state (green) and with PTX in the uncoupled intermediate state (gold, PDB ID: 8OQA), depicted as in *e*.

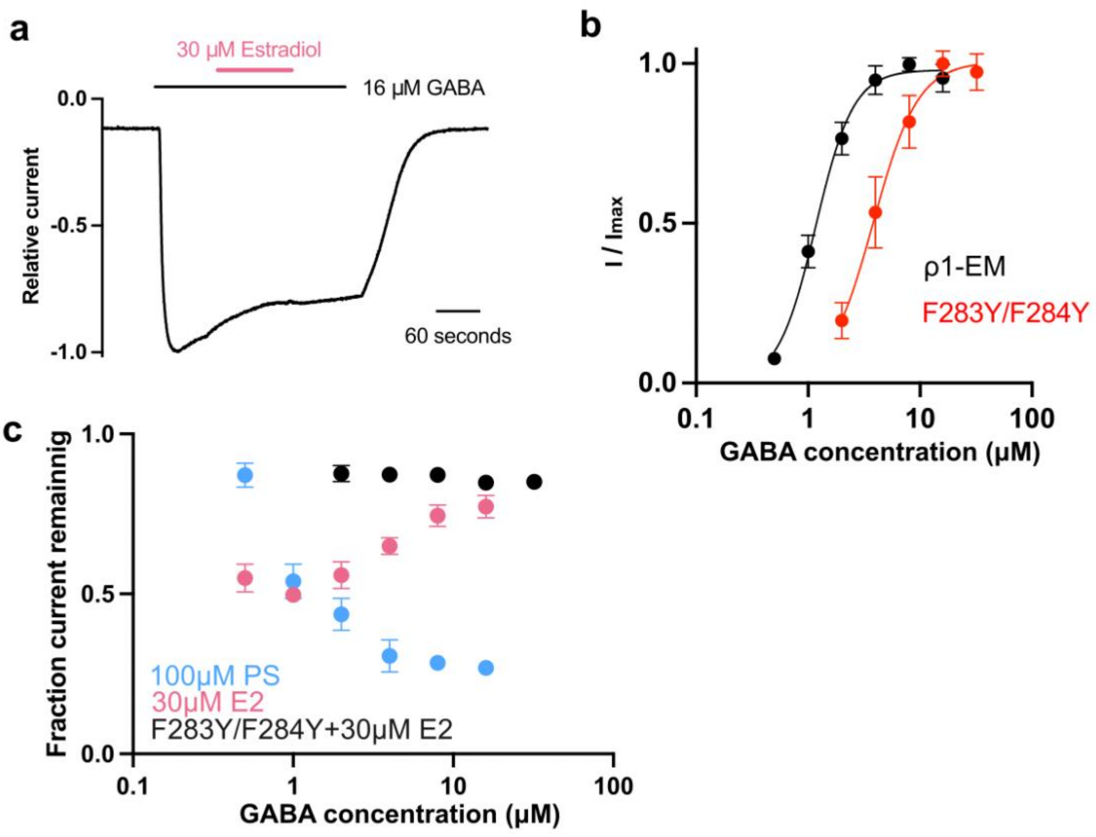

Supplementary Figure 5. Pore-radius and electrophysiology profiles of ρ1-EM relevant to the E2 site.

(a) Sample trace from TEVC recording of ρ1-EM in response to a saturating GABA concentration (16 μM) alone or in combination with 30 μM E2 as indicated at top of plot.

(b) GABA concentration response curves for ρ1-EM wild-type (black) and F283Y/F284Y (red) variants. Error bars represent SEM for 8 individual oocytes for ρ1-EM or 3 oocytes for the mutant. Solid lines represent fits to Boltzmann curves with an EC_{50} of 1.2 μM (wild-type, 95% confidence interval 1.04-1.32 μM) or 3.8 μM (F283Y/F244Y, 95% confidence interval 2.93-5.38 μM).

(c) Fractional current remaining after treatment with 30 μM E2 (pink) or 100 μM PS (blue) of the ρ1-EM response to GABA, plotted against co-applied GABA concentrations. Black points represent fractional current remaining of the F283Y/F284Y after treatment with 30 μM E2. Error bars represent SEM, derived from 5 individual oocytes for wild-type ρ1-EM with 30 μM E2, or 3 individual oocytes for the other two conditions.

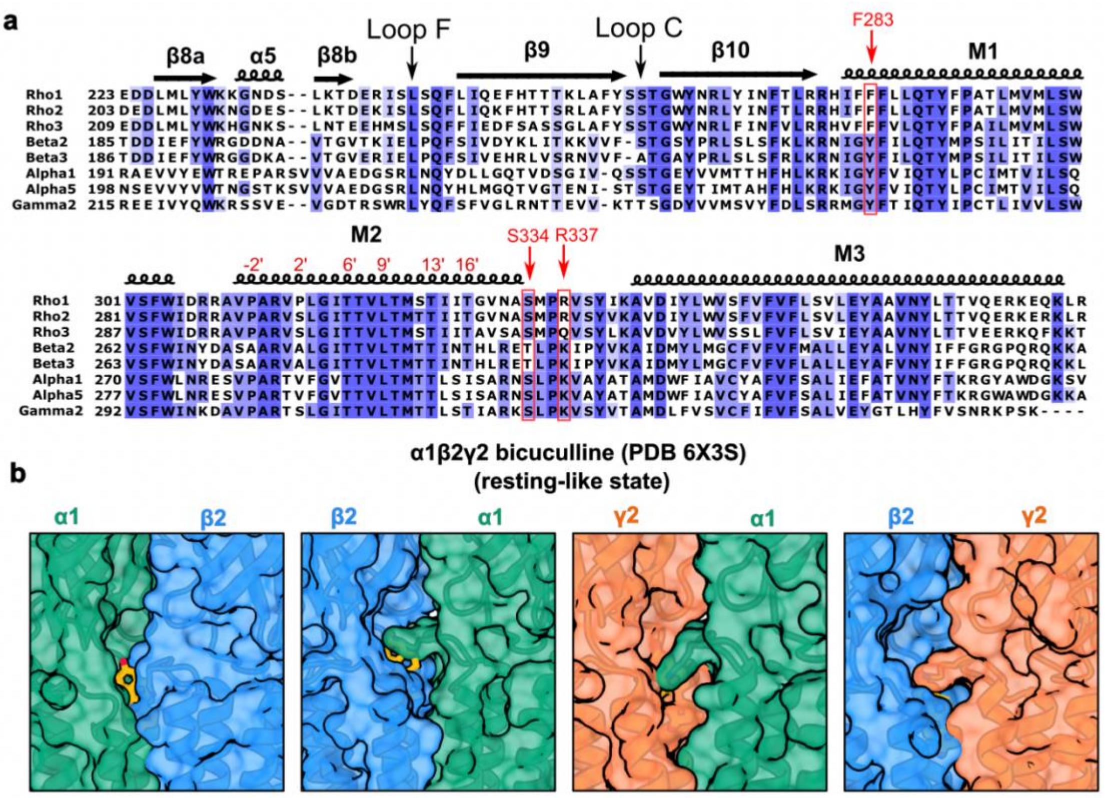

Supplementary Figure 6. Sequence and structure comparisons among GABA$_A$-receptor subtypes around the E2 binding site.

(a) Sequence alignment of representative human ρ, β, α and γ2 GABA$_A$ receptor subunits. Residues are numbered according to reference UniProt sequences. Key structural features are labeled above alignment, including secondary-structure elements and agonist-binding loops C and F. Red labels indicate TMD residues interacting with E2, and M2 pore-facing residues in prime notation. For simplicity, only residues between β8 and M3 relevant to steroid binding in this work are shown.

(b) Surface representations, depicted as in *Supplementary Figure 4a,* showing zoom views of the four types of subunit interfaces in a previously reported cryo-EM structure of the α1β2γ2 GABA$_A$ receptor (PDB ID: 6X3S). The structure contains the competitive inhibitor bicuculline and is assigned, like ρ1-EM with E2, to a resting-like state. Superimposition of E2 (yellow) from the corresponding ρ1-EM structure indicates little capacity for binding in this site.

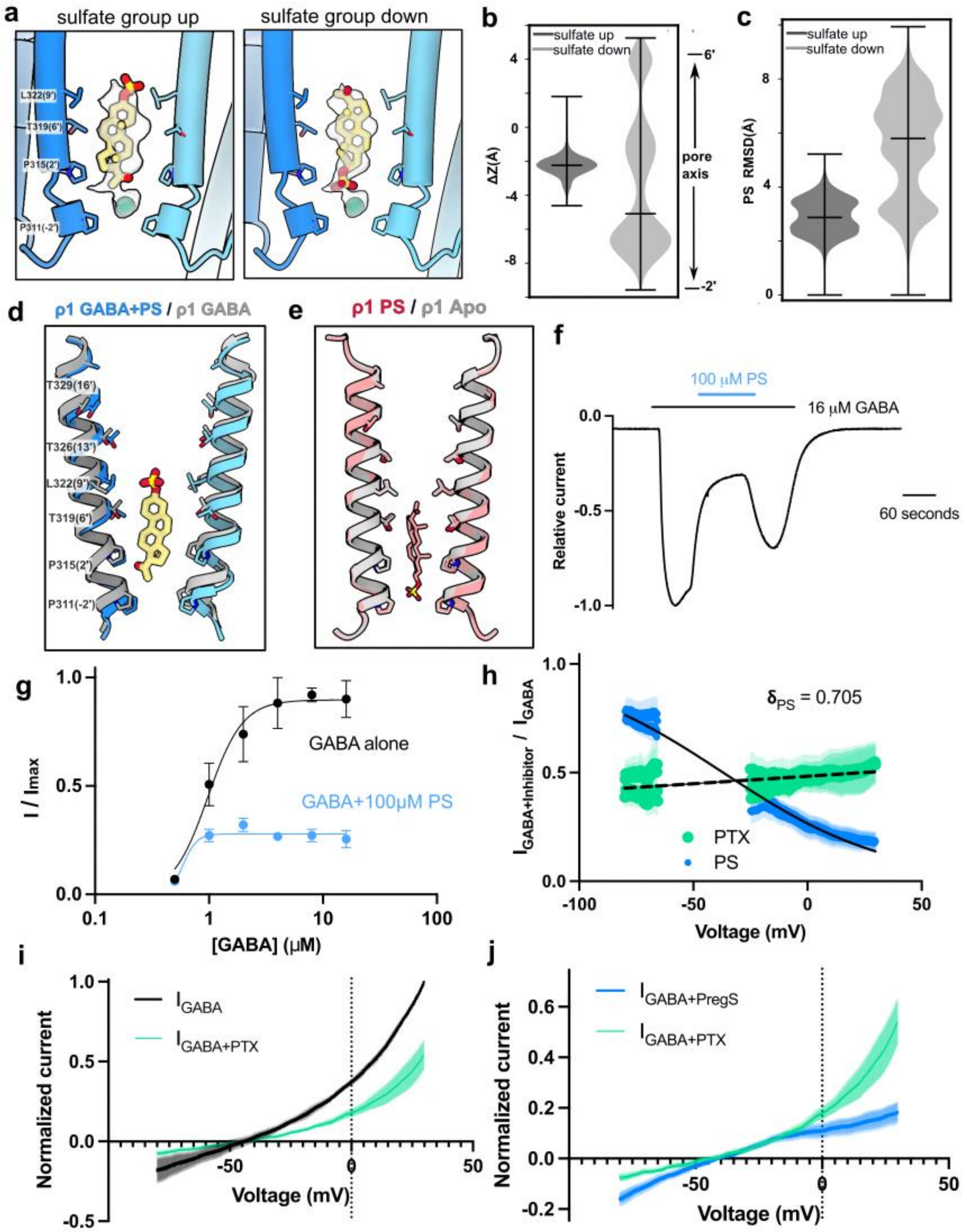

a
sulfate group up
sulfate group down
L322(9')
T319(6')
P315(2')
P311(-2')
b
sulfate up
sulfate down
ΔZ(Å)
6'
-2'
pore axis
c
sulfate up
sulfate down
PS RMSD(Å)
d
ρ1 GABA+PS / ρ1 GABA
T329(16')
T326(13')
L322(9')
T319(6')
P315(2')
P311(-2')
e
ρ1 PS / ρ1 Apo
f
100 μM PS
16 μM GABA
Relative current
60 seconds
g
I / Imax
GABA alone
GABA+100μM PS
[GABA] (μM)
h
IGABA+Inhibitor / IGABA
δPS = 0.705
PTX
PS
Voltage (mV)
i
Normalized current
IGABA
IGABA+PTX
Voltage (mV)
j
Normalized current
IGABA+PregS
IGABA+PTX
Voltage (mV)

Supplementary Figure 7. MD simulations and electrophysiology of ρ1-EM relevant to the PS site.

(a) Zoom views of the inner pore of ρ1-EM determined with GABA and PS, with experimental density assigned to PS and chloride shown in transparency. Two possible poses are shown for PS, either with the sulfate group oriented up towards the 9' hydrophobic gate (left) or down towards the cytosol (right). PS (yellow), chloride (green) and surrounding residues are shown as sticks and labeled.

(b) Translocation of PS along the pore z-axis in MD simulations launched from the two poses shown in *A*. Simulation frames are aligned on Cα atoms of the M2 pore-lining helices, and translocation (ΔZ) calculated for the center of mass of PS non-hydrogen atoms along a linear axis passing through the channel pore. Violin plots represent probability densities from 4 independent simulation replicates of >400 ns each, sampled every 0.4 ns (n = 4443 and 4132 for simulations with the sulfate oriented up and down, respectively), with markers indicating median and extrema.

(c) Mobility of PS calculated from RMSD of PS non-hydrogen atoms, with sample sizes and plot parameters as in *b*.

(d) Superimposition of two opposing M2 helices in structures of GABA-bound ρ1-EM in the absence (gray, PDB ID: 8OP9) and presence (blue) of PS.

(e) Superimposition of two opposing M2 helices in structures of ρ1-EM in the absence (gray, PDB ID: 8OQ6) and presence (red) of PS.

(f) Sample trace from TEVC recording of ρ1-EM in response to saturating GABA (16 μM), alone or in combination with 100 μM PS as indicated in bars at top of plot.

(g) GABA concentration response curves in the absence (black) and presence of 100 μM PS (blue). Error bars represent SEM from 5 individual oocytes. Solid lines represent fits to Boltzmann curves with an EC_{50} of 1.0 μM (GABA alone, 95% confidence interval 0.74-1.36 μM) or 0.6 μM (GABA+PS). Fit for the GABA+PS curve is not converged and included only for comparison. However, GABA response is saturated by 1 μM in the presence of PS indicating a decreased EC50 relative to the GABA only curve.

(h) Fractional current remaining of 1 μM GABA responses upon co-application with 100 μM PS (blue) or 500 nM PTX (green), plotted against membrane potential. Shaded region represents SEM for 4 individual oocytes. Points within 15 mV of the reversal potential were removed due to large errors associated with division by values near 0. Data are fit to the Woodhull model with the fitting parameters: δ=0.705 (95% CI 0.692 to 0.719) and K_D^{0mV}=36.3 (95% CI 35.6 to 37.0 μM) for PS.

(i) Background-subtracted and normalized current-voltage curves for voltage ramps in the presence of GABA alone (black) or in combination with 500 nM PTX (green). Shaded regions represent SEM from 4 individual oocytes.

(j) Overlay of current-voltage responses to GABA co-applied with PTX (green) or PS (blue) normalized to the maximum response to GABA alone. Data reproduced from Figure 3H and panel (i) in this figure.

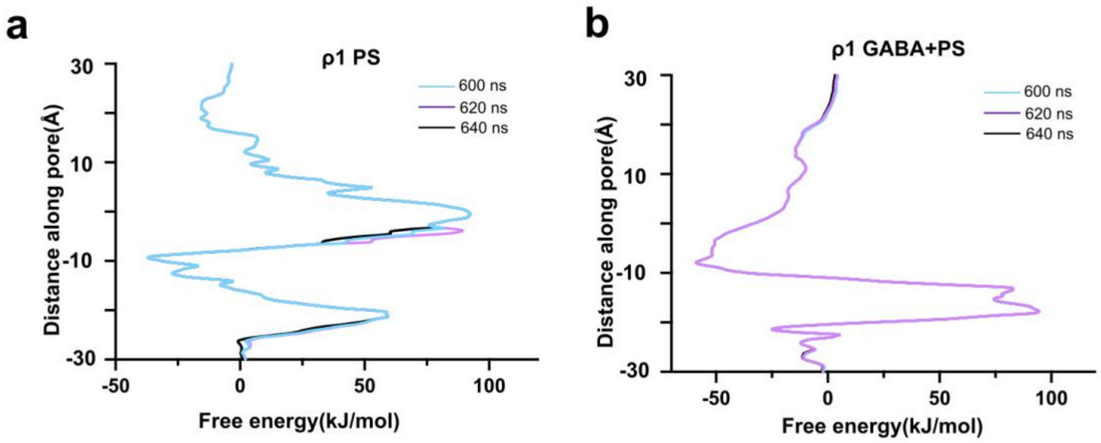

Supplementary Figure 8. Convergence of potential of mean force (PMF) calculations.

(a) PMF free energy curves at multiple MD simulation time points for PS movement along the pore axis in the PS-bound structure of ρ1-EM in the absence of GABA.

(b) PMF free energy curves at multiple MD simulation time points for PS movement along the pore axis in the PS-bound structure of ρ1-EM in the presence of GABA.
