# Supplementary material for: Divergent mechanisms of steroid inhibition in the human ρ1 GABAA receptor
Source: Nat Commun. 2024 Sep 6;15:7795. doi: 10.1038/s41467-024-51904-7 (PMC11379708; doi:10.1038/s41467-024-51904-7)
Supplement: Supplementary file 3 — Reporting Summary [file 41467_2024_51904_MOESM3_ESM.pdf]

nature portfolio

Corresponding author(s): Rebecca J. Howard, Erik Lindahl

Last updated by author(s): 26 July 2024

Reporting Summary

Nature Portfolio wishes to improve the reproducibility of the work that we publish. This form provides structure for consistency and transparency in reporting. For further information on Nature Portfolio policies, see our Editorial Policies and the Editorial Policy Checklist.

Statistics

For all statistical analyses, confirm that the following items are present in the figure legend, table legend, main text, or Methods section.

n/a	Confirmed	
☐	☒	The exact sample size (n) for each experimental group/condition, given as a discrete number and unit of measurement
☐	☒	A statement on whether measurements were taken from distinct samples or whether the same sample was measured repeatedly
☐	☒	The statistical test(s) used AND whether they are one- or two-sided<br>*Only common tests should be described solely by name; describe more complex techniques in the Methods section.*
☒	☐	A description of all covariates tested
☒	☐	A description of any assumptions or corrections, such as tests of normality and adjustment for multiple comparisons
☐	☒	A full description of the statistical parameters including central tendency (e.g. means) or other basic estimates (e.g. regression coefficient) AND variation (e.g. standard deviation) or associated estimates of uncertainty (e.g. confidence intervals)
☐	☒	For null hypothesis testing, the test statistic (e.g. F, t, r) with confidence intervals, effect sizes, degrees of freedom and P value noted<br>*Give P values as exact values whenever suitable.*
☒	☐	For Bayesian analysis, information on the choice of priors and Markov chain Monte Carlo settings
☒	☐	For hierarchical and complex designs, identification of the appropriate level for tests and full reporting of outcomes
☒	☐	Estimates of effect sizes (e.g. Cohen's d, Pearson's r), indicating how they were calculated

Our web collection on statistics for biologists contains articles on many of the points above.

Software and code

Policy information about availability of computer code

Data collection	EPU 3.5.0 (cryo-EM), pCLAMP 10 (electrophysiology)
Data analysis	Relion 3.1.4, CTFFIND 4.1, topaz 0.2.5, CryoSPARC v4.2.1, PHENIX 1.18.2, Coot 0.9.5, CHAP 0.9.1, UCSF ChimeraX 1.3, CHARMM-GUI, GROMACS 2021.5, VMD 1.9.3

For manuscripts utilizing custom algorithms or software that are central to the research but not yet described in published literature, software must be made available to editors and reviewers. We strongly encourage code deposition in a community repository (e.g. GitHub). See the Nature Portfolio guidelines for submitting code & software for further information.

Data

Policy information about availability of data

All manuscripts must include a data availability statement. This statement should provide the following information, where applicable:

- Accession codes, unique identifiers, or web links for publicly available datasets
- A description of any restrictions on data availability
- For clinical datasets or third party data, please ensure that the statement adheres to our policy

Cryo-EM maps and corresponding atomic coordinates have been deposited in the Electron Microscopy Data Bank (EMDB) and the Protein Data Bank (PDB) respectively for structures with E2 (EMD-19167, PDB-8RH4), GABA+E2 in the primed state (EMD-19171, PDB-8RH7), GABA+E2 in the desensitized state (EMD-19172, PDB-8RH8), PS (EMD-19173, PDB-8RH9), and GABA+PS (EMD-19175, PDB-8RHG). MD simulation trajectories and parameter files are available on Zenodo (https://doi.org/10.5281/zenodo.10406748).

Research involving human participants, their data, or biological material

Policy information about studies with human participants or human data. See also policy information about sex, gender (identity/presentation), and sexual orientation and race, ethnicity and racism.

Reporting on sex and gender	This research did not involve human participants, their data, or biological material.
Reporting on race, ethnicity, or other socially relevant groupings	This research did not involve human participants, their data, or biological material.
Population characteristics	This research did not involve human participants, their data, or biological material.
Recruitment	This research did not involve human participants, their data, or biological material.
Ethics oversight	This research did not involve human participants, their data, or biological material.

Note that full information on the approval of the study protocol must also be provided in the manuscript.

Field-specific reporting

Please select the one below that is the best fit for your research. If you are not sure, read the appropriate sections before making your selection.

[x] Life sciences [ ] Behavioural & social sciences [ ] Ecological, evolutionary & environmental sciences

For a reference copy of the document with all sections, see nature.com/documents/nr-reporting-summary-flat.pdf

Life sciences study design

All studies must disclose on these points even when the disclosure is negative.

Sample size	No statistical method was used to predetermine sample sizes. Electrophysiology measurements were performed in 3–8 independent oocytes, with exact sample sizes indicated in respective legends; these sample sizes were found to enable distinction between conditions beyond standard errors. MD simulations were assessed on the basis of frames sampled each ns, for a total of 300–400 frames per condition, also indicated in figure legends, and also enabling ≥2-fold distinctions in variability. The number of cryo-EM particles used to determine each structure are shown in Table 1, indicating they are sufficient to generate maps at the reported resolutions (≤3.8 Å).
Data exclusions	No electrophysiology or MD data were excluded. For cryo-EM, exclusion criteria were not pre-established. 2D and 3D classification yielded multiple classes, of which only those that showed clear structural signal were selected for final reconstruction and refinement.
Replication	All experiments were reproduced in multiple samples. Electrophysiology measurements were reproduced in at least 3 individual oocytes. MD simulation trajectories were replicated 4 times for each system. For cryo-EM experiments, multiple rounds of refinement were performed, resulting in visually equivalent maps. No results were observed to lack reproducibility.
Randomization	For electrophysiology and MD simulations, as repeated measures were performed with indistinguishable oocytes and compute hardware, no randomization was performed. For structure determination, particles in each dataset were split randomly into two groups, and the gold-standard Fourier shell correlation cutoff was applied to determine overall resolution.
Blinding	According to standard practices in electrophysiology, MD simulations, and structure determination, no blinding was deemed necessary, as the measured variables are not substantially subjective or subject to investigator influence.

Reporting for specific materials, systems and methods

We require information from authors about some types of materials, experimental systems and methods used in many studies. Here, indicate whether each material, system or method listed is relevant to your study. If you are not sure if a list item applies to your research, read the appropriate section before selecting a response.

Materials & experimental systems

n/a	Involved in the study
x	Antibodies
	x Eukaryotic cell lines
x	Palaeontology and archaeology
x	Animals and other organisms
x	Clinical data
x	Dual use research of concern
x	Plants

Methods

n/a	Involved in the study
x	ChIP-seq
x	Flow cytometry
x	MRI-based neuroimaging

Eukaryotic cell lines

Policy information about cell lines and Sex and Gender in Research

Cell line source(s)	Expi293F GnTI- cells, originally derived from kidney cells of a female human fetus, were purchased from Gibco (cat #A39240). Sf9 TriEX cells, originally derived from ovarian tissue of a female armyworm, were purchased from Novagen (cat #71023).
Authentication	Cell lines were freshly purchased and not tested further.
Mycoplasma contamination	No mycoplasma contamination was detected.
Commonly misidentified lines (See ICLAC register)	No commonly misidentified lines were used.

Plants

Seed stocks	*Report on the source of all seed stocks or other plant material used. If applicable, state the seed stock centre and catalogue number. If plant specimens were collected from the field, describe the collection location, date and sampling procedures.*
Novel plant genotypes	*Describe the methods by which all novel plant genotypes were produced. This includes those generated by transgenic approaches, gene editing, chemical/radiation-based mutagenesis and hybridization. For transgenic lines, describe the transformation method, the number of independent lines analyzed and the generation upon which experiments were performed. For gene-edited lines, describe the editor used, the endogenous sequence targeted for editing, the targeting guide RNA sequence (if applicable) and how the editor was applied.*
Authentication	*Describe any authentication procedures for each seed stock used or novel genotype generated. Describe any experiments used to assess the effect of a mutation and, where applicable, how potential secondary effects (e.g. second site T-DNA insertions, mosiacism, off-target gene editing) were examined.*
